# SNX-BAR proteins 5 and 6 are required for NCOA7-AS antiviral activity against influenza A virus

**DOI:** 10.1016/j.jbc.2026.113372

**Published:** 2026-07-28

**Authors:** Mary Arnaud-Arnould, Antoine Rebendenne, Marine Tauziet, Serge Urbach, Khadija El Koulali, Emiliano P. Ricci, Mélanie Wencker, Olivier Moncorgé, Mickaël Blaise, Caroline Goujon

**Affiliations:** 1 IRIM, University of Montpellier, CNRS, Montpellier, France; 2 LBMC, INSERM, CNRS, ENS-Lyon, Lyon University, Lyon, France; 3 CIRI, INSERM, CNRS, ENS-Lyon, Lyon University, Lyon, France; 4 IGF, University of Montpellier, CNRS, INSERM, Montpellier, France; 5 BCM, University of Montpellier, CNRS, INSERM, Montpellier, France

**Keywords:** influenza A virus, NCOA7-AS, SNX, V-ATPase, restriction

## Abstract

Interferon-induced antiviral proteins act on the first line of defense against viruses, including influenza A virus (IAV). Among those, the short isoform of nuclear receptor coactivator 7 (NCOA7-AS) has been shown to inhibit IAV entry and more especially the fusion between endosomal and viral membranes. Ectopic expression of NCOA7-AS leads to the increased acidification of the endolysosomal pathway, putatively through NCOA7-AS interaction with the vacuolar ATPase (V-ATPase). However, the precise mechanism of action of NCOA7-AS is not fully understood. Here, we identified the cellular partners of NCOA7-AS by mass spectrometry, including the V-ATPase subunits known to interact with NCOA7-AS. A point mutation disrupting the interaction with the V-ATPase led to loss of antiviral activity against IAV, demonstrating that V-ATPase engagement is required for the antiviral phenotype. Moreover, sorting nexin (SNX) 1/2/5/6 were identified as novel partners of NCOA7-AS. These proteins are involved in retrograde transport of cellular cargoes from endosomes to the trans-Golgi network. We revealed that SNX5/6 interacted directly with NCOA7-AS and were essential for NCOA7-AS antiviral activity against IAV. Interestingly, crystal structures of NCOA7-AS/SNX5 complexes showed that the SNX5-interaction motif in NCOA7-AS was similar to those found in known cargoes of SNX5/6. In addition, we could pinpoint several critical residues that were important for binding and antiviral activity. Collectively, our study identifies novel essential partners for NCOA7-AS antiviral activity and the structural basis for their interaction.

Seasonal influenza A viruses (IAV) pose a major threat to global health, putting significant pressure on healthcare systems and causing significant morbidity and mortality each year, according to the World Health Organization (https://cdn.who.int/media/docs/default-source/influenza/report-of-the-first-global-consultation-november-2009.pdf). IAV are respiratory viruses, belonging to the *Orthomyxoviridae* family, that cause mild to moderate upper respiratory tracts illnesses. Successful infection of host cells by IAV depends on a complex, multistep entry process. The hemagglutinin (HA) protein of human-adapted strains of IAV interacts preferentially with glycoconjugates bearing terminal α-2,6-linked sialic acids (reviewed in (1)). Following this, IAV particle is internalized through endocytosis (2). This early step requires numerous host factors involved in the endocytic pathway, as well as the vacuolar ATPase (V-ATPase), which drives the progressive acidification of the endosomal lumen (3). The V-ATPase is a multi-subunit complex, composed of two distinct domains: the membrane-associated V0 domain, which is responsible for proton translocation to the lumen of the vesicle, and the cytoplasmic V1 domain, which provides energy by ATP hydrolysis (reviewed in (4)). Endosomal acidification by the V-ATPase is critical for HA acidification, fusion peptide exposure and subsequent HA-mediated fusion between viral and endosomal membranes. Furthermore, luminal acidification activates the viral M2 ion channel, leading to proton influx and acidification within the viral particle (5, 6, 7, 8). This induces conformational changes in the matrix protein M1, disrupting the viral capsid and the interaction between M1 and the viral ribonucleoproteins complexes (vRNPs), leading to the subsequent release of vRNPs in the cytoplasm (9, 10).

IAV entry is a critical step targeted by many potent antiviral proteins, including some coded by interferon-stimulated genes that are part of the interferon (IFN) system. The IFNs act as one of the first lines of defense against viral infections (reviewed in (11, 12)). Notably, the 219-amino acid, IFN-inducible, short isoform of nuclear receptor coactivator 7 (NCOA7), termed NCOA7-Alternative-Start (NCOA7-AS; also referred to as NCOA7 isoform 4 or NCOA7-B; (13)), has been shown to inhibit IAV entry (14) as well as endosome-mediated coronavirus entry (15, 16). In contrast to this IFN-inducible, antiviral isoform, the longer NCOA7 isoform (herein referred to as NCOA7-FL) is constitutively expressed and has been primarily implicated in neuronal and lysosomal homeostasis (14, 17, 18). NCOA7-FL and NCOA7-AS belong to the Tre2/Bub2/Cdc16 (TBC), lysin motif (LysM), domain catalytic (TLDc)-containing family of proteins. Despite their name, no enzymatic activity has ever been reported for TLDc-bearing proteins (19), but they have been shown to protect cells from oxidative stress, by an unknown mechanism (20). Interestingly, the TLDc proteins are known interactors of the V-ATPase (18, 21). Ectopic expression of NCOA7-AS increases acidification of the endolysosomal pathway, supposedly through NCOA7-AS interaction with the V-ATPase (14). NCOA7-AS ectopic expression is also associated with a decrease in IAV/endolysosomal membrane fusion (14). However, the precise molecular mechanism of action of NCOA7-AS is yet to be unraveled.

Here, we employed mass spectrometry to identify the cellular partners of NCOA7-AS. This approach confirmed NCOA7-AS’ interaction with multiple V-ATPase subunits. Disruption of this interaction by a single amino acid substitution abolished antiviral activity against IAV, underscoring the importance of V-ATPase engagement in mediating the antiviral effect. In addition, sorting nexins (SNXs) SNX1, SNX2, SNX5 and SNX6 were identified as novel binding partners of NCOA7-AS. These proteins are known to play roles in retrograde transport of cellular cargoes to the trans-Golgi network (22). We further demonstrated that SNX5 and SNX6 bind directly to NCOA7-AS’ amino-terminal domain and were required for IAV inhibition and the overacidification of the endolysosomal compartment. Moreover, crystal structures of NCOA7-AS in complex with SNX5 revealed that the SNX5-binding motif in NCOA7-AS resembles those found in established SNX5/6 cargoes (23). Collectively, these findings strongly suggest that NCOA7-AS uses SNX proteins to suppress IAV replication.

## Results

### The interaction between NCOA7-AS and V1 subunits of the V-ATPase is required for antiviral activity

**Figure 1:**
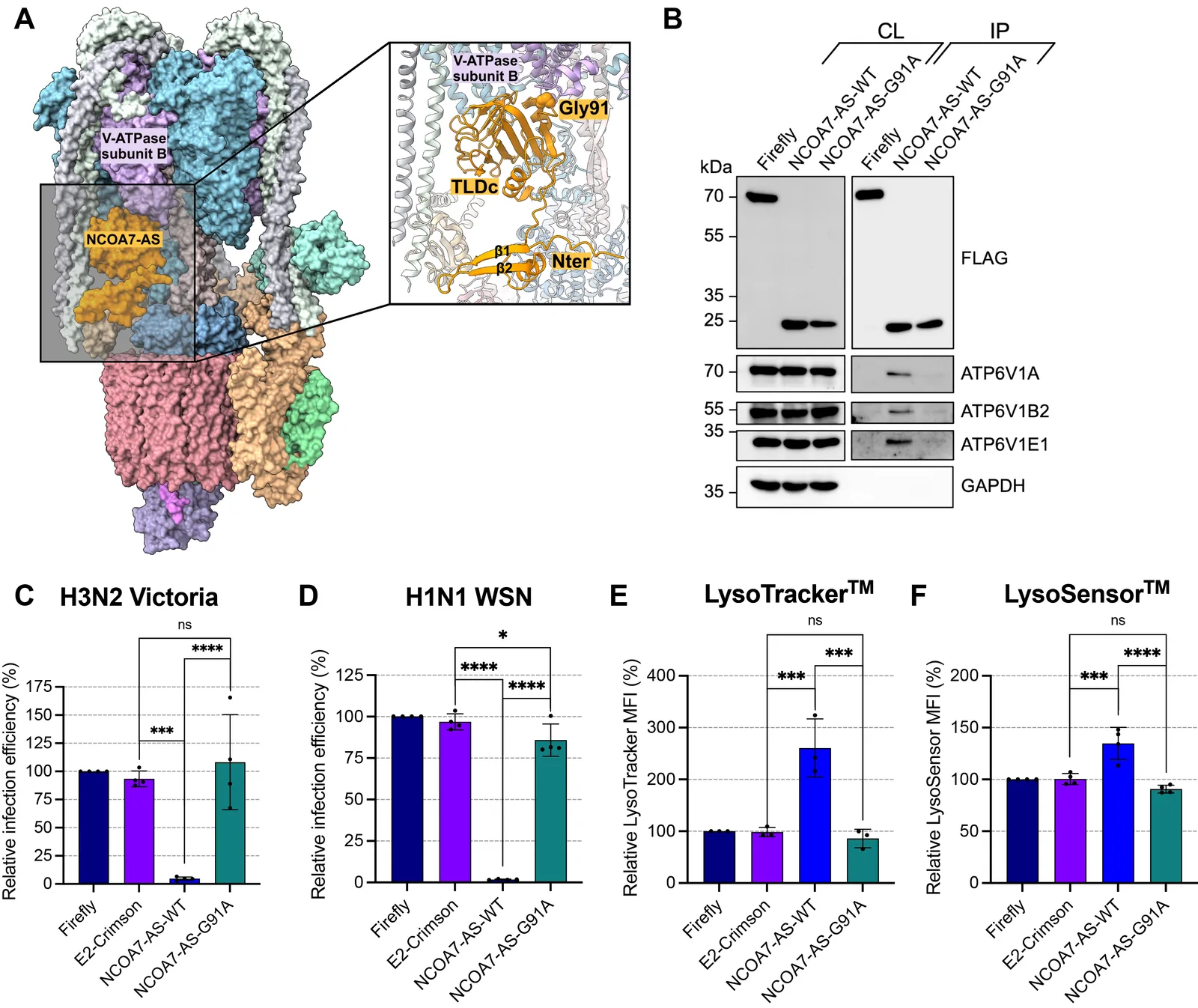
**NCOA7-AS G91 is critical for V-ATPase binding, antiviral activity against IAV, and endolysosomal overacidification.***A*, docking of NCOA7-AS AlphaFold model on the Cryo-EM structure of human V-ATPase bound to mEAK7, a TLDc domain containing protein (PDB:7unf). The NCOA7-AS model (in *orange*) was superposed onto the TLDc domain of mEAK7 using ChimeraX. The zoom panel indicates that the loop harboring NCOA7-AS G91, shown as *spheres*, is in close proximity to the V-ATPase B subunit. *B*–*F*, A549 cells were transduced with lentiviral vectors to stably express FLAG-tagged Firefly (a negative control), -E2-Crimson (another negative control), -NCOA7-AS-WT or -NCOA7-AS-G91A. *B*, cells were lysed and the FLAG-tagged proteins immunoprecipitated and subsequently analyzed by immunoblotting using the indicated antibodies; GAPDH served as a loading control (a representative immunoblot is shown out of three independent experiments). *C* and *D*, cells were infected with IAV strains encoding a nanoluciferase reporter gene: either A/Victoria/3/75 at MOI 0.5 (*C*) or A/WSN/1933 at MOI 0.25 (*D*). The relative infection efficiency was measured by monitoring Nanoluciferase activity 8 h postinfection. *E* and *F*, cells were incubated with LysoTracker Green DND-26 (*E*) or with LysoSensor Green DND-189 (*F*) for 1 h at 37 °C and the mean fluorescence intensity (MFI) was subsequently analyzed by flow cytometry. *C*–*F*, results were normalized to 100% for the control condition (Firefly), and the mean and SD of three independent experiments are shown; one-way ANOVA. IAV, influenza A virus; NCOA7-AS, -nuclear receptor coactivator 7-alternative start; TLDc, TBC/LysM-associated domain-catalytic; V-ATPase, vacuolar ATPase.

TLDc proteins are known interactors of the V-ATPase (18, 21). Notably, both NCOA7-FL and NCOA7-AS, which share a C-terminal TLDc domain, have been shown to interact with V1 subunits of the V-ATPase (ATP6V1B1/2 and also A, C and G) and to promote endolysosomal acidification in cells (14, 17, 18). As previously shown, several residues within TLDc proteins are highly conserved across species and are critical for V-ATPase binding (21). Notably, mutations of glycine 815, glycine 845 and glycine 896 in mouse NCOA7-FL abolished interaction with the V-ATPase (21). Among these three residues, only glycine 815 is exposed on the surface of the TLDc, therefore the focus was made on the equivalent glycine in NCOA7-AS (glycine 91). As shown by docking of NCOA7-AS AlphaFold model on the Cryo-EM structure of human V-ATPase bound to another TLDc domain containing protein, mEAK7 (24), this glycine 91 is present on a short loop in close proximity to the V-ATPase subunit B (Fig. 1*A*). In order to study its importance, glycine 91 was mutated to an alanine (G91A). Lung-derived A549 cells were transduced to stably express a negative control (Firefly), wild-type NCOA7-AS (NCOA7-AS-WT) or NCOA7-AS-G91A mutant. Co-immunoprecipitation experiments confirmed that NCOA7-AS interacted with V1 subunits of the V-ATPase, namely ATP6V1A, ATP6V1B2 and ATP6V1E1, while NCOA7-AS-G91A mutant was unable to interact with these subunits (Fig. 1*B*).

To determine whether interactions of NCOA7-AS with the V-ATPase were indeed required for antiviral activity and overacidification of the endolysosomal compartment, NCOA7-AS-G91A antiviral activity was tested against nanoluciferase-encoding influenza reporter viruses A/Victoria/3/75 (H3N2) and A/WSN/1933 (H1N1) in single-cycle experiments. Ectopic expression of NCOA7-AS-WT led to a strong inhibition of A/Victoria/3/75 and A/WSN/1933 infection (Fig. 1, *C* and *D*), while NCOA7-AS-G91A mutant showed almost no antiviral activity. Furthermore, the endosomal/lysosomal compartment size and relative acidification levels were assessed using LysoTracker and LysoSensor reagents, respectively. This confirmed that NCOA7-AS-WT induced a significant expansion of the acidic compartments (Fig. 1*E*) and a decrease in endosomal/lysosomal pH (Fig. 1*F*). In contrast, NCOA7-AS-G91A mutant had no impact as the values were similar to the negative control conditions, strongly arguing that the interaction of NCOA7-AS with the V-ATPase is required for antiviral activity against IAV and overacidification of the endolysosomal compartment.

### SNX5 and SNX6 are cellular partners of NCOA7-AS required for its function

**Figure 2:**
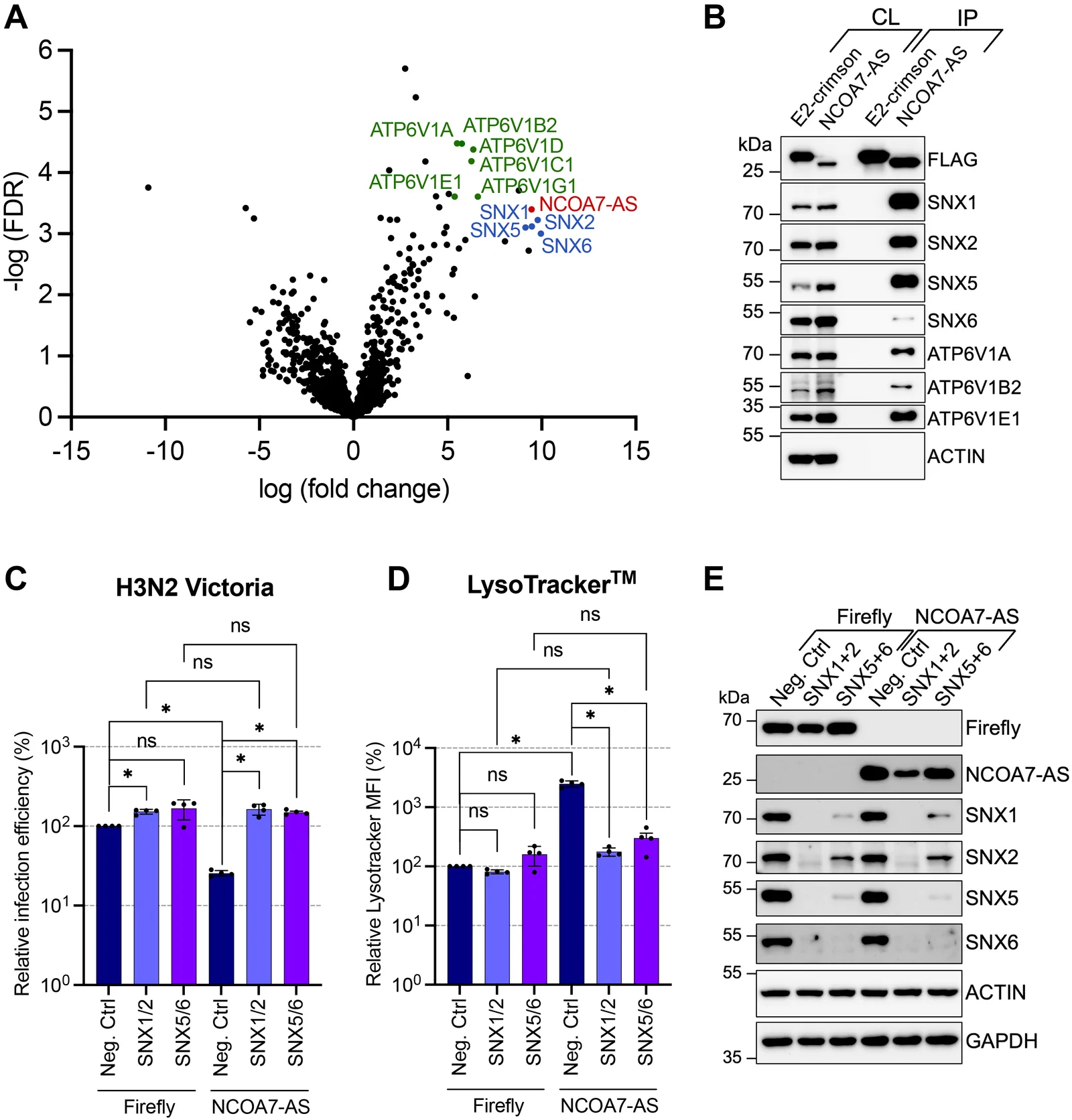
**SNX1, SNX2, SNX5and SNX6 act as physical and functional partners of NCOA7-AS.***A*, Volcano Plot of differentially abundant proteins between NCOA7-AS and control immunoprecipitation, presenting the fold change (in log2) and significance (−Log 10 *p* value) obtained from a *t* test based on intensity values from three independent experiments. *B*, U87-MG cells were transduced with lentiviral vectors expressing FLAG-tagged-NCOA7-AS or -E2-Crimson (a negative control). Cells were lysed and the FLAG-tagged proteins were immunoprecipitated and subsequently analyzed by immunoblotting using the indicated antibodies; Actin served as a loading control. *C*–*E*, A549 cells were transduced with an all-in-one CRISPRi system, expressing dual nontargeting sgRNAs (Neg. Ctrl) or dual sgRNAs targeting either SNX1/2 or SNX5/6, and then further transduced to express either FLAG-tagged-Firefly or -NCOA7-AS. Cells were infected with IAV (A/Victoria/3/75) encoding a Nanoluciferase reporter gene at an MOI of 0.25 and the relative infection efficiency was measured by monitoring Nanoluciferase activity 8 h later (*C*). In parallel, cells were incubated with LysoTracker Green DND-26 for 1 h at 37 °C and the MFI was subsequently analyzed by flow cytometry (*D*). SNX depletion in the cells used in *C* and *D* was assessed by immunoblotting using the indicated antibodies; Actin and GAPDH served as loading control (*E*). Representative immunoblots (out of three independent experiments) are shown (*B* and *E*) and the mean and SD of three independent experiments (*C* and *D*); Mann-Whitney test on raw data (*C*) or on log-transformed raw data (*D*). NCOA7-AS, nuclear receptor coactivator 7-alternative start; SNX, sorting nexin; IAV, influenza A virus.

To identify additional binding partners of NCOA7-AS and gain further insight into its mode of action, 3xFLAG-tagged NCOA7-AS was stably expressed in U87-MG glioblastoma cells, which are highly permissive to lentiviral (LV) transduction and support high levels of transgene expression. The protein was then immunoprecipitated, and the resulting complexes were analyzed by mass spectrometry (Table S1). The interaction between NCOA7-AS and several V1 subunits of the V-ATPase, namely ATP6V1A, ATP6V1B2, ATP6V1C1, ATP6V1D, ATP6V1E1, and ATP6V1G1, was confirmed (Fig. 2*A* and Table S1). The NCOA7-AS interactome additionally contained four members of the SNX family, SNX1, SNX2, SNX5 and SNX6, which were particularly enriched (Fig. 2*A* and Table S1).

SNX proteins are important for retrograde transport of proteins between endosomes and the trans-Golgi network (22). They notably contain a Phox homology (PX) domain, and some of them are known to interact with phosphoinositides. Hence PX-SNX1 and PX-SNX2 interact with phosphatidylinositol-3-phosphate at the endosomal membrane (25, 26, 27, 28) but not PX-SNX5 and PX-SNX6 (29). Moreover, SNX1/2/5/6 belong to the SNX-Bin-Amphiphysin-Rvs (BAR) subfamily, which possesses a BAR domain. The BAR domain is important for dimerization and to detect and/or induce membrane curvature (30, 31, 32). A heterodimer of SNX-BAR proteins, consisting of SNX1 or SNX2 associated with either SNX5 or SNX6, forms the retromer complex in association with a vacuolar protein sorting trimer. The Retromer complex mediates cargo trafficking from the endosome to the trans-Golgi network (33). However, recent studies have shown that some cargoes could be transported by a SNX1/2-SNX5/6-dependent pathway that was Retromer-independent (34, 35, 36). Interestingly, an additional, noncanonical role has also been reported for SNX5. Indeed, SNX5 has been shown to inhibit several viruses, including IAV, by inducing autophagy at virus-containing endosomes independently of SNX1, SNX2 or the Retromer complex (37).

Interactions between NCOA7-AS and SNX1, SNX2, SNX5 and SNX6, identified by mass spectrometry, were validated by co-immunoprecipitation, with all four endogenous SNX proteins co-immunoprecipitating with FLAG-tagged NCOA7-AS-WT but not with the E2-Crimson control in U87-MG cells (Fig. 2*B*).

To assess the role of SNX1, SNX2, SNX5 and SNX6 in NCOA7-AS antiviral activity against IAV and in the overacidification of the endolysosomal compartment, these proteins were depleted in A549 cells using dual CRISPRi targeting SNX1/2 or SNX5/6, alongside a dual nontargeting control. As mentioned previously, SNX1/2 and SNX5/6 function redundantly (35) and double knockdowns were therefore used to circumvent the redundancy. The depleted cells were transduced with LV vectors to stably express a control (Firefly) or NCOA7-AS. Depletion of either SNX1/SNX2 or SNX5/SNX6 fully restored viral infection in the presence of NCOA7-AS, while they modestly enhanced IAV infection in its absence (Fig. 2*C*). Consistently, the ability of NCOA7-AS to enhance endolysosomal acidification was abolished upon depletion of either SNX1/2 or SNX5/6 (Fig. 2*D*).

Immunoblot analyses confirmed efficient depletion of the targeted proteins (Fig. 2*E*). Notably, each double knockdown affected the stability of the corresponding heterodimer, consistent with previous findings (35). This effect was more pronounced upon SNX1/2 depletion: while residual SNX1 and SNX2 remained detectable in SNX5/6-depleted cells, SNX5 and SNX6 were no longer detectable in SNX1/2-depleted cells. Moreover, SNX1/2 depletion also impacted the expression levels of Firefly and NCOA7-AS, an effect that was not observed, or only marginal, upon SNX5/6 depletion.

Taken together, these results indicate that these SNX-BAR proteins are required for NCOA7-AS-mediated endolysosomal overacidification and antiviral activity against IAV. However, the contribution of SNX1/2 should be interpreted with caution, as their depletion seemed to affect NCOA7-AS expression levels, whereas the requirement for SNX5/6 appeared more direct.

### The N-terminal domain (NTD) of NCOA7-AS directly interacts with the Phox domain of SNX5 and SNX6

SNX5 and SNX6 interact with more than 60 cargoes, including cation-independent mannose-6-phosphate receptor (CI-MPR) (35) or *Chlamydia trachomatis* IncE (38, 39, 40, 41). These interactions are mediated by the PX domain of SNX5 or SNX6 (PX-SNX5 and PX-SNX6). Analysis of the sequence requirement to bind SNX5 or SNX6 revealed that cargoes have a conserved secondary structure, consisting of two antiparallel β-strands with one β-strand containing a ΦxΩxΦ motif, where Φ is a hydrophobic residue, x, any amino acid and Ω, an aromatic residue (23).

NCOA7-AS contains two main structural domains: the NTD of 53 amino acids and the TLDc domain, the latter being involved in the interaction with the V-ATPase (21). The structure of the human NCOA7-AS TLDc domain has been recently determined (42), but that of NCOA7-AS-NTD was unknown. AlphaFold prediction suggested however that NCOA7-AS-NTD could be organized as two antiparallel β-strands and a small α-helix (Fig. S1*A*). Furthermore, the first predicted β-strand contains a ΦxΩxΦ motif (Fig. S1*B*), suggesting that NCOA7-AS could directly interact with SNX5 or SNX6.

**Figure 3:**
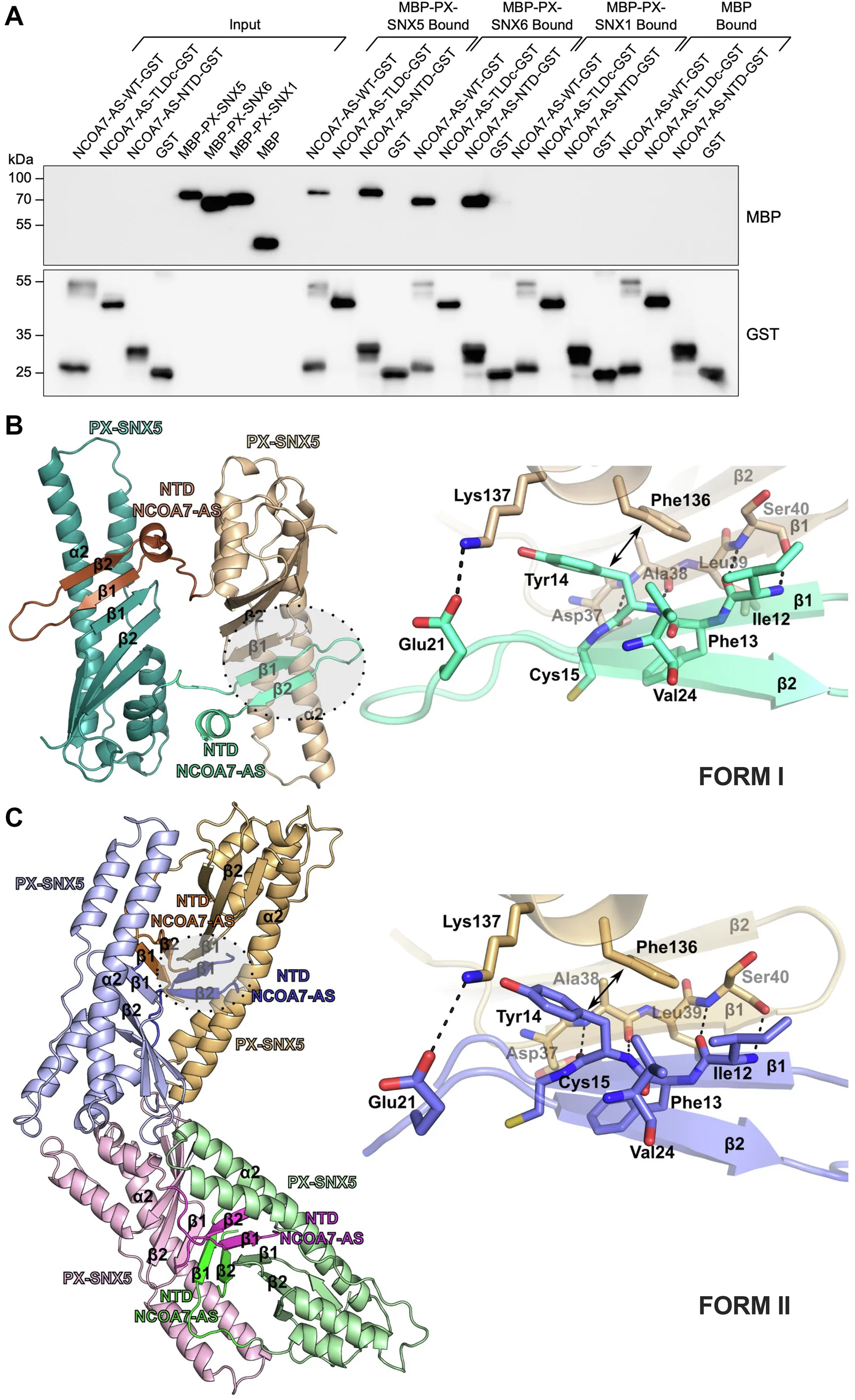
**The N-terminal domain (NTD) of NCOA7-AS interacts directly with the PX domain of SNX5 and SNX6.***A*, recombinant MBP-tagged-PX-SNX5, -PX-SNX6, -PX-SNX1 or MBP alone, and GST-tagged-NCOA7-AS-WT, -NCOA7-AS-NTD, -NCOA7-AS-TLDc or GST alone, were produced in *Escherichia coli* and purified. GST pull-down assays were performed using these recombinant proteins and analyzed by Western blot. *B* and *C*, crystal structure of PX-SNX5-NCOA7-AS-NTD chimera (PDB: 9IGY). Two crystal forms were solved. In the form I (*B*), the asymmetric unit contains one monomer, the dimer interaction described in here is therefore formed with the symmetric molecule. The second crystal form (*C*) contains four molecules per asymmetric unit and therefore contains two dimers. The overall structures are shown in cartoon representations in *B* and *C*, for all the structures, the NCOA7-AS-NTD is a darker color as compared to the PX-SNX5 domain fused to it. The *right panel* shows a magnified view of the region, rotated 180° relative to the highlighted zone in *gray*. The H-bonds interaction between the strands β1 of NCOA7-AS and β1 of the PX domain are shown in *black dashed lines*. The salt-bridge between Glu21 and Lys137 is also represented by a *dashed line*. The main interaction between the side chain of Tyr14 from NCOA7-AS and Phe136 of the PX domain is illustrated by the *double headed arrows*. Val24 also contributes to interact with Phe136, but the interaction is not shown. NCOA7-AS, nuclear receptor coactivator 7-alternative start; PX, Phox homology; SNX, sorting nexin; TLDc, TBC/LysM-associated domain-catalytic.

To test this hypothesis and to map the regions of NCOA7-AS potentially important for SNX binding, the proteins and domains of interest were individually produced in *Escherichia coli* as fusions with Glutathione S-Transferase (GST) or Maltose Binding Protein (MBP) and purified, in order to perform *in vitro* GST pull-down assays. GST-tagged-NCOA7-AS (NCOA7-AS-WT-GST), -NCOA7-AS NTD (amino acids 1-53; NCOA7-AS-NTD-GST), -NCOA7-AS TLDc domain (amino acids 54-219, NCOA7-AS-TLDc-GST) and MBP-tagged-PX-SNX1, -PX-SNX5, -PX-SNX6 were used. The GST pull-downs showed that NCOA7-AS interacted with the PX domains of SNX5 and SNX6 but not with the PX domain of SNX1 (Fig. 3*A*). As previously described by other groups, the PX domains of SNX5 and SNX6 contain a unique 38 amino acid insertion, which folds in a helix-turn-helix extension, that is absent in the PX domains of SNX1 and SNX2 (23). This insertion has already been shown to be important for the interaction with several cargoes, such as CI-MPR (23) and IncE (38, 39, 40, 41).

Furthermore, the ability of NCOA7-AS to bind to the PX domain of SNX5 and SNX6 was mediated by the NTD and not by the TLDc, as shown by the fact that, contrary to NCOA7-AS-NTD-GST, NCOA7-AS-TLDc-GST was not able to bind PX-SNX5 and PX-SNX6 (Fig. 3*A*). Altogether, these results showed that NCOA7-AS directly interacted with SNX5 and SNX6 PX domains *via* its N-terminal domain.

In order to better understand the molecular basis of this interaction, we employed a strategy previously described by another team to determine the structure of the two interacting partners (40). This strategy consists in designing a chimeric fusion protein with the two binding partners. Such a strategy was successfully used to obtain the crystal structure of PX-SNX5 in complex with a peptide from CI-MPR (23). Therefore, a chimeric protein composed of PX-SNX5 (amino acids 25-172) and NCOA7-AS N-terminal sequence (amino acids 4-36) was produced. After recombinant expression in *E. coli* and purification (Fig. S2*A*), the chimeric protein was crystallized into two different forms (named forms I and II). The X-ray structures of the two distinct crystals forms were solved at high resolution (Fig. 3, *B* and *C* and Table S2).

PX-SNX5 domain is typically composed of three β-strands (β1, β2 and β3) and three α-helices (α1, α2 and α3) as seen in a previously reported structure (PDB: 5wy2; (41)). The N-terminus of NCOA7-AS forms a β-hairpin structure consisting of β1 (residues Gln11-Cys15) and β2 strands (residues Phe23-Ile27) and a short α-helix (residues Val29-Arg35). The β-hairpin structure of NCOA7-AS-NTD interacts with PX-SNX5 by forming an anti-parallel β-sheet. The β1 strand of NCOA7-AS strongly interacts by H-bonds with the β-sheet through β1 of the PX-SNX5 domain, and with its complementary groove at the base of the extended α-helical insertion (α2) of the PX-SNX5 domain. The N-terminal β1 strand of NCOA7-AS forms the primary interaction interface with SNX5 through backbone hydrogen bonds between its β1 strand and the β1 strand of the SNX5 PX domain. The two anti-parallel β-strands of NCOA7-AS are connected by a short loop (Ala16-Pro22) that could not be rebuilt in the crystal form II, however, this loop is not making any direct contact with the SNX5 protein in the other form (form I). The NCOA7-AS β1 strand forms an interface of interaction with the extended α-helical region (α2) of the PX-SNX5 domain. This interaction is mediated by several van der Waals interactions but the strongest interaction is driven by the side chain of Tyr14 from β1 strand of NCOA7-AS, through a hydrophobic interaction with the side chain of Phe136 of SNX5. To a lesser extent, Val24 side chain of NCOA7-AS interacts as well with Phe136 of SNX5. A salt-bridge interaction is also established between PX domain Lys137 and NCOA7-AS Glu21. Of note, although the two crystals forms are packing differently and notably with a different conformation of the NCOA7-AS hairpin in both structures, the β strands interaction and the main interaction between NCOAS-AS Tyr14 and SNX5 Phe136 are conserved in both structures. This is strongly supportive of a specific interaction not driven by the chimera or the crystal packing.

Furthermore, PX-SNX5’s mode of interaction with NCOA7-AS was also compared with the previously solved structures of PX-SNX5 bound to other proteins, namely Sema4C (PDB: 6n5z; (23), CI-MPR (PDB: 6n5y; (23) and IncE (PDB: 5wy2; (41) (Fig. S2*B*). This analysis demonstrated that NCOA7-AS interacted in a similar manner as the other interactors of SNX5, by forming an anti-parallel β-sheet and through a hydrophobic interaction between the Phe136 of PX and either a Tyr or a Phe for the different binding partners. Of note, PX-SNX6 is thought to harbor the same binding specificity to cargoes through its Phe137.

**Figure 4:**
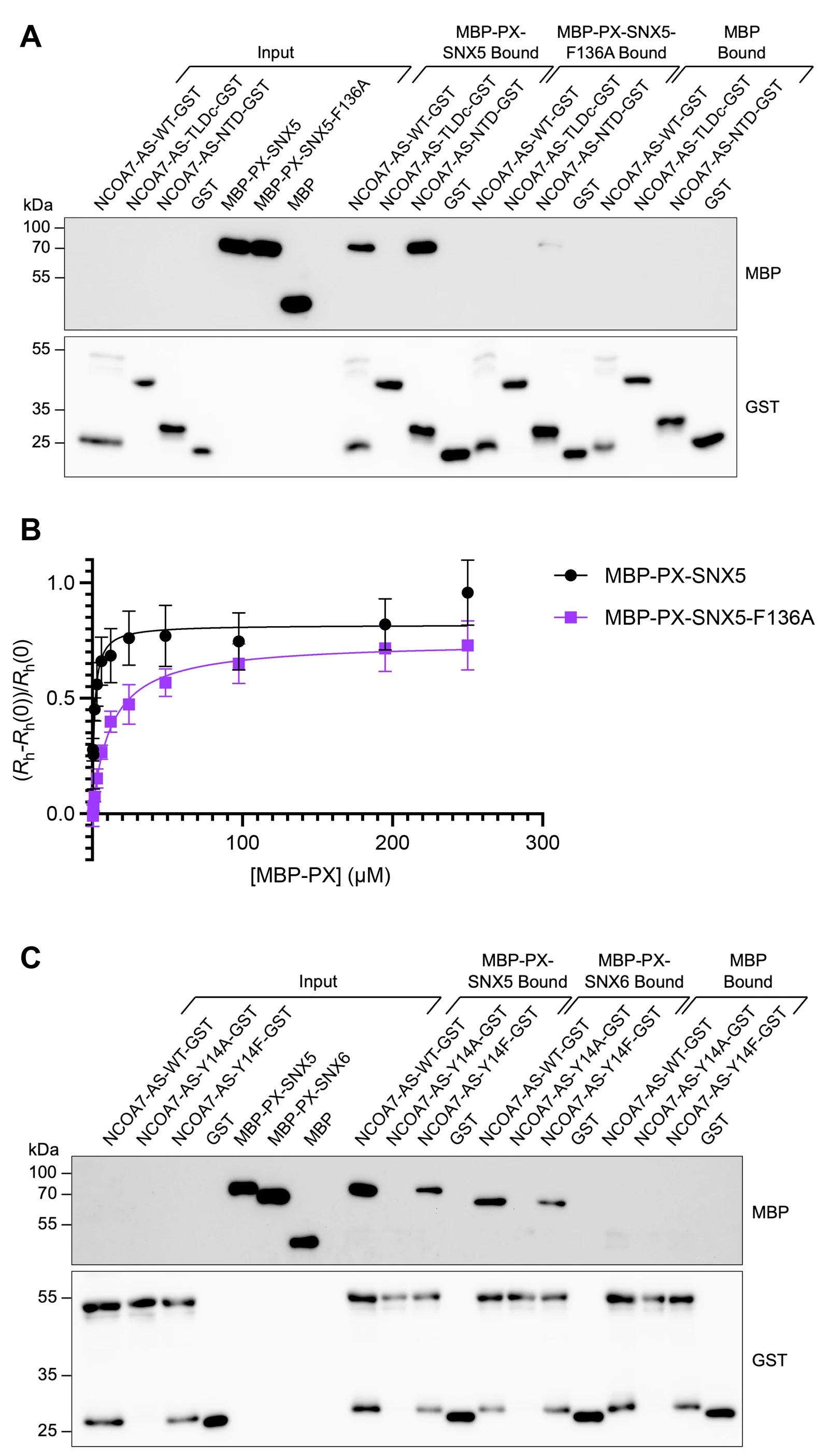
**NCOA7-AS-Y14 and SNX5-F136 are important for NCOA7-AS/SNX5 and NCOA7-AS/SNX6 interaction.***A*, recombinant MBP-tagged-PX-SNX5, -PX-SNX5-F136A or MBP alone, and GST-tagged-NCOA7-AS-WT, -NCOA7-AS-NTD, -NCOA7-AS-TLDc or GST alone were produced in *Escherichia coli* and purified. GST pull-down was performed using these recombinant proteins and analyzed by Western blot. *B*, microfluidic diffusional sizing of MBP-tagged-PX-SNX5 or -PX-SNX5-F136A with fluorescently labeled NCOA7-AS-NTD (NCOA7-AS-NTD-FITC) was measured using Fluidity One-W. Values represent the means and SD of three independent experiments. *C*, recombinant MBP-tagged-PX-SNX5, -PX-SNX6 or MBP alone and GST-tagged-NCOA7-AS-WT, -NCOA7-AS-Y14A, -NCOA7-AS-Y14F or GST alone were produced in *Escherichia coli* and purified. GST pull-down was performed using these recombinant proteins and analyzed by western blot. NCOA7-AS, nuclear receptor coactivator 7-alternative start; NTD, N-terminal domain; PX, Phox homology; SNX, sorting nexin; TLDc, TBC/LysM-associated domain-catalytic.

To confirm the importance of these residues for the specific interaction seen in the two crystal structures, Phe136 was mutated into Ala (F136A) on MBP-PX-SNX5. *In vitro* GST pull-down assays with NCOA7-AS-WT-GST demonstrated that PX-SNX5 Phe136 was important for NCOA7-AS binding to SNX5 (Fig. 4*A*). To gain further insight into this interaction, the apparent dissociation constant (*K*d) between the PX-SNX5 domain and the N-terminus of NCOA7-AS was determined using microfluidic diffusional sizing (MDS) (43, 44, 45, 46) (Fig. 4*B*). Microfluidic diffusional sizing enables determination of the hydrodynamic radius (*R*_h_) of macromolecules based on their diffusion properties in solution. Because macromolecular complexes diffuse more slowly than unbound particles, complex formation results in an increase of the apparent *R*_h_. The measured signal therefore reflects the average *R*_h_ of all particles present in solution. At binding saturation, the observed *R*_h_ is maximal, whereas in the absence of interaction the *R*_h_ remains minimal. To monitor these changes, the fluorescently labeled binding partner, typically the smaller molecule, is tracked during the experiment.

In this set-up, the *R*_h_ of fluorescently labeled NCOA7-AS peptide corresponding to its NTD was estimated in solution in the absence or the presence of increased concentrations of MBP-PX-SNX5 or MBP-PX-SNX5-F136A domains. In the absence of ligand, NCOA7-AS peptide possesses an *R*_h_ of about 1.6 nm, while it reaches 3.1 and 2.7 nm at the highest concentration (250 μM) of WT and mutant PX-SNX5, respectively. The titration curve strongly attests that the single point mutation F136A significantly increases the apparent (*K*d) between PX-SNX5 and NCOA7-AS NTD (*K*d=1.4 ± 0.6 μM for the PX-SNX5-WT *versus* 13.4 ± 4.6 μM for the F136A mutant). The analysis of the impact of the reciprocal mutation of Tyr14 to Ala on NCOA7-AS (NCOA7-AS-Y14A) confirmed the importance of this residue for the *in vitro* binding to PX-SNX5 (Fig. 4*C*). Of note, NCOA7-AS Val24 mutation to Ala was also tested. *In vitro* GST pull-down assays showed that the interaction between this mutant and PX-SNX5 was slightly decreased but not abolished (data not shown), contrary to what was observed with the Y14A mutant (Fig. 4*C*).

Moreover, Tyr14 was mutated to a minimal conservative Phe substitution, which maintained the aromatic cycle in the side chain. Y14F mutation in NCOA7-AS did not affect its ability to bind to PX-SNX5 and SNX6 (Fig. 4*C*), highlighting the fact that the hydrophobic interaction mediated by Tyr14 and Phe136 drives the interaction between NCOA7-AS and SNX5 or SNX6.

### Residue Tyr14 on NCOA7-AS is essential for NCOA7-AS-mediated acidification of endolysosomes and antiviral activity against IAV

**Figure 5:**
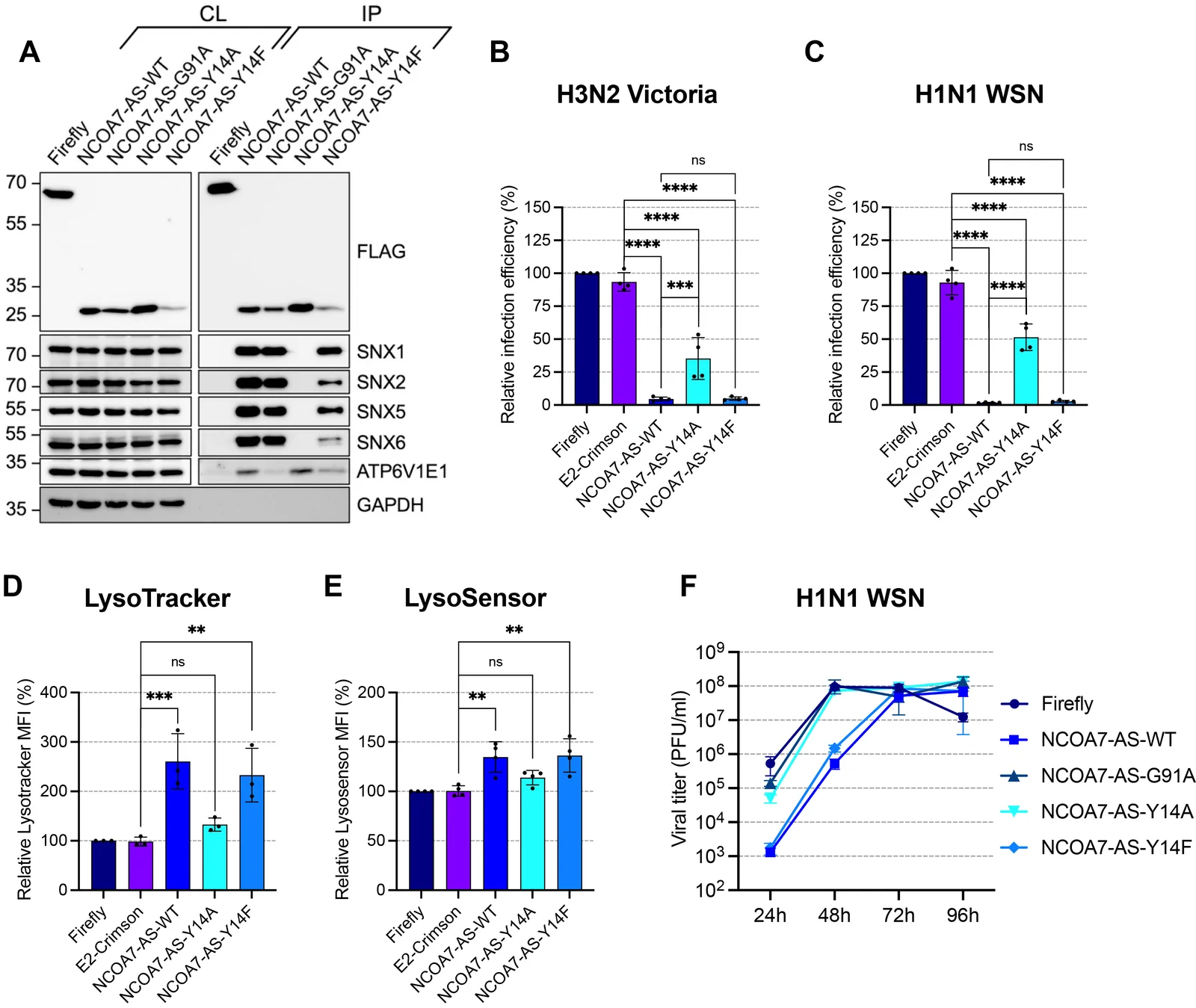
**NCOA7-AS Y14 is required for SNX5 binding, antiviral activity against IAV, and endolysosomal overacidification.***A*–*F*, A549 cells stably expressing FLAG-tagged-Firefly (a negative control), -E2-Crimson (another negative control), -NCOA7-AS-WT, -NCOA7-AS-G91A, -NCOA7-AS-Y14A or -NCOA7-AS-Y14F were generated. *A*, cells expressing the indicated proteins were lysed and the FLAG-tagged proteins immunoprecipitated, followed by immunoblotting analysis using the indicated antibodies; GAPDH served as a loading control. *B* and *C*, cells expressing the indicated proteins were infected with Nanoluciferase-expressing A/Victoria/3/75 at MOI 0.5 (*B*) or A/WSN/1933 at MOI 0.25 (*C*). The relative infection efficiency was measured by monitoring nanoluciferase activity at 8 h postinfection. *D* and *E*, cells expressing the indicated proteins were incubated with LysoTracker Green DND-26 (*D*) or with LysoSensor Green DND-189 (*E*) for 1 h at 37 °C and the MFI subsequently analyzed by flow cytometry. *F*, cells expressing the indicated proteins were infected with A/Victoria/3/75 at MOI 0.005 in multicycle replication conditions; supernatants were harvested at the indicated time points post infection and infectious virus production was measured by plaque assays on MDCK cells. Data represent the mean and SD of 4 (*B* and *C*) or 3 (*D* and *E*) independent experiments (one-way ANOVA) or show one representative experiment with the mean and SDs of technical triplicates (*F*). IAV, influenza A virus; MDCK, Madin-Darby canine kidney; NCOA7-AS, nuclear receptor coactivator 7-alternative start.

To assess the importance of NCOA7-AS-Y14 in the antiviral activity against IAV, point mutations of NCOA7-AS-Y14 were generated, and A549 cells stably expressing a negative control (Firefly), NCOA7-AS-WT, NCOA7-AS-G91A, NCOA7-AS-Y14A or NCOA7-AS-Y14F were generated. Co-immunoprecipitation experiments first confirmed that NCOA7-AS-Y14A mutant did not interact with any of the 4 SNXs of interest. Minimal conservative NCOA7-AS-Y14F mutant conserved the interaction with SNX5 (and the other SNXs), confirming that the interaction between NCOA7-AS and SNX5 requires π-stacking between NCOA7-AS-Y14 and SNX5-F136 (Fig. 5*A*).

Although NCOA7-AS-G91A failed to interact with the V-ATPase—as evidenced by the loss of binding to the ATP6V1E1 subunit (Figs. 1*B* and 5*A*)—it retained interaction with SNX5 and other SNX proteins. This indicates that NCOA7-AS association with SNXs is independent of its interaction with the V-ATPase, consistent with the direct binding observed *in vitro* (Fig. 4).

Next, we tested the antiviral activity of these mutants against IAV in single-cycle infection experiments. Compared to NCOA7-AS-WT, NCOA7-AS-Y14A was poorly functional with a significant reduction of antiviral activity against both A/Victoria/3/75 (Fig. 5*B*) and A/WSN/1933 (Fig. 5*C*). In contrast, NCOA7-AS-Y14F retained full antiviral activity despite being less expressed at steady state than NCOA7-AS-WT. The use of LysoTracker and LysoSensor showed that NCOA7-AS-Y14A had no impact on endolysosomal acidification, whereas NCOA7-AS-Y14F was as active as NCOA7-AS-WT (Fig. 5, *D* and *E*).

To further validate these results, multicycle infection experiments were performed with A/WSN/1933 in A549 cells stably expressing a negative control (Firefly), NCOA7-AS-WT, NCOA7-AS-G91A, NCOA7-AS-Y14A or NCOA7-AS-Y14F (Fig. 5*F*). Both NCOA7-AS-WT and NCOA7-AS-Y14F inhibited IAV infection by two logs at 24 h and 48 h, while NCOA7-AS-G91A and NCOA7-AS-Y14A had no impact on viral growth (similarly to the Firefly negative control). From 72 h onwards, the antiviral effect of these proteins was not observed anymore, suggesting that their impact might be transient, saturable, or overcome over time.

Taken together, these data showed that residue Tyr14 on NCOA7-AS, which mediated the interaction with SNX5 and SNX6, was essential for antiviral activity against IAV and for inducing the endolysosomal overacidification.

## Discussion

In this study, we identify key determinants of NCOA7-AS activity against IAV and its impact on endolysosomal pH. Analysis of the NCOA7-AS interactome revealed SNX1, SNX2, SNX5 and SNX6 as novel binding partners, which contributed to NCOA7-AS–mediated overacidification of endolysosomes and antiviral activity. However, the respective contributions of SNX1/2 and SNX5/6 were not equivalent. While depletion of either dimer impaired NCOA7-AS function, interpretation of SNX1/2 involvement was limited by indirect effects on NCOA7-AS expression levels. In contrast, SNX5/6 appeared to play a clearer and more direct role, consistent with their direct interaction with NCOA7-AS.

We were able to characterize in depth, biochemically and structurally, the interaction between SNX5/6 and NCOA7-AS. First, both full length NCOA7-AS and its N-terminal domain interacted directly with the PX domain of SNX5 and SNX6, as shown using recombinant proteins. Such a direct interaction was not observed with SNX1, highlighting the primary role of SNX5/6 in NCOA7-AS function. We solved the X-ray structure of the PX-SNX5 domain bound to the N-terminal domain of NCOA7-AS, which enabled us to map the interface of interaction between the two partners. This showed a binding mode that was similar to the other cargoes of SNX5 (23, 41). The N-terminal domain of NCOA7-AS interacted with the PX domain of SNX5 *via* the formation of a β-sheet with strands β1, β2, and β3 of SNX5 in a complementary groove at the base of the extended α-helical insertion (α2) of the PX-SNX5 domain. This region corresponds to the 38 amino acid insertion in the PX domain that is uniquely present in SNX5 and SNX6, as well as their homologous protein SNX32, but not found in SNX1 and SNX2. This observation presumably explains why we could not detect a direct interaction between NCOA7-AS and SNX1. Notably, the PX domain of SNX32 exhibits a high sequence homology with the PX domains of SNX5 and SNX6. However, SNX32 was not found in the interactome of NCOA7-AS, suggesting that it does not play a role in NCOA7-AS activity. This could be due to subtle differences in SNX32 sequence or subcellular localization as compared to SNX5 and SNX6, and would require further exploration.

The structure showed that the hydrophobic interaction mediated by SNX5 F136 and NCOA7-AS Y14, facing each other, seemed to be crucial. The essential role of these residues in the NCOA7-AS/SNX5 interaction was validated *in vitro*. The importance of NCOA7-AS Y14 was further confirmed in cells for the binding to SNX5, the overacidification phenotype induced by NCOA7-AS as well as its antiviral activity.

We also identified G91 within the TLDc domain of NCOA7-AS as a critical residue mediating its interaction with the V1 subunits of the V-ATPase. This interaction is essential for both the overacidification of the endosomal compartment and the antiviral activity against IAV. Hence, our current model proposed that ectopic expression of NCOA7-AS disrupts the finely tuned pH gradient of the endolysosomal pathway through its interaction with the V-ATPase, thereby impairing IAV entry. V-ATPase activity is regulated through multiple mechanisms, including the dynamic assembly and disassembly of the V0/V1 complex (reviewed in (47)). Intriguingly, the Cryo-EM structure of the V-ATPase in complex with another TLDc protein, yeast OXR1, revealed that OXR1 inhibits V-ATPase activity by promoting complex disassembly (48). Consistently, the TLDc domains of human OXR1 and NCOA7-AS have also been shown to inhibit V-ATPase activity *in vitro* by inducing disassembly (49). These findings contrast with our own data. However, the *in vitro* study relied on purified, recombinant TLDc domains, which may not fully recapitulate the regulatory complexity present in a cellular context with the full-length proteins. Supporting this, a previous study showed that the long isoform of NCOA7 (NCOA7-FL) enhanced V-ATPase assembly and activity *in vivo* in mice (17). Moreover, in human cells, NCOA7-AS ectopic expression clearly increases V-ATPase activity ((14) and this study). The apparent discrepancy between *in vitro* and *in vivo*/*ex vivo* data likely reflects the contribution of additional domains in NCOA7-AS and NCOA7-FL that influence the outcome of the TLDc–V-ATPase interaction. In support of this, we found that NCOA7-AS binding partners SNX1/2/5/6 are required for the overacidification of the endolysosomal compartment driven by NCOA7-AS. These findings underscore the importance of binding partners in modulating the activity of TLDc-containing proteins and add an additional layer of complexity to the regulation of V-ATPase function in cells. Whether SNX5/6 are primarily required to ensure the correct subcellular localization of NCOA7-AS or to recruit additional factors involved in regulating V-ATPase activity remains to be determined.

Together, these findings shed new light on the molecular mechanism of action of NCOA7-AS, defined novel essential cellular partners, as well as crucial residues for its antiviral activity.

## Experimental procedures

### Plasmids

The pRRL.sin.cPPT.SFFV/FLAG-E2-Crimson-IRES-PuromycinR.WPRE, pRRL.sin.cPPT.SFFV/FLAG-Firefly-IRES-PuromycinR.WPRE and pRRL.sin.cPPT.SFFV/FLAG-NCOA7-AS-IRES-PuromycinR.WPRE have been previously described (14, 50). Of note, FLAG-E2-Crimson and FLAG-Firefly are used as irrelevant, negative controls expressed from the same backbone as NCOA7-AS, as previously (3, 14, 51). pRRL.sin.cPPT.SFFV/FLAG-NCOA7-AS-G91A-IRES-PuromycinR.WPRE, pRRL.sin.cPPT.SFFV/FLAG-NCOA7-AS-Y14A-IRES-PuromycinR.WPRE and pRRL.sin.cPPT.SFFV/FLAG-NCOA7-AS-Y14F-IRES-PuromycinR.WPRE were obtained by site-directed mutagenesis (overlap-extension PCR).

To express the C-terminal GST-tag version of NCOA7-AS recombinant proteins, a new pET30-MCS-cleavage site for tobacco etch virus (TEV) protease-GST CDS-His6 CDS was created from the pET-30 Ek/LIC expression vector (Novagen). The NCOA7-AS full length, TLDc domain, NTD of NCOA7-AS, NCOA7-AS-Y14A and NCOA7-AS-Y14F CDS were PCR amplified from the aforementioned pRRL.sin.cPPT.SFFV/IRES-puro.WPRE LV vector plasmid and cloned into the modified pET-30 expression vector using EcoRI and HindIII cloning sites.

For CRISPRi-mediated knockdown, ZIM3 KRAB domain (52) was fused to the N-terminus of a nuclease-dead Cas9 from plentidCas9-VP64_blast (Addgene, #61425) and cloned into the pLentiCRISPRv2 backbone (Addgene, #52961), where the original sgRNA scaffold was replaced by a new version (Addgene, #133459), generating the pLentiCRISPRi-v3 vector.

CRISPRi LVs coding for sgRNAs targeting SNX1, SNX2, SNX5, SNX6 and control sgRNAs were obtained by cloning annealed oligonucleotides in BsmBI-digested pLentiCRISPRi-v3, as described (Addgene). sgRNAs targeting SNX1, SNX2, SNX5, SNX6, were designed with CRISPick. The sgRNA coding sequences used were as follow: Ctrl neg g#1 5′- AGCACGTAATGTCCGTGGAT-3′, Ctrl neg g#2 5′- CAATCGGCGACGTTTTAAAT-3′, SNX1 g#1 5′- CGGGTGGAAGAAGATGGCGT -3′, SNX1 g#2 5′- TGGTGGTGGCTGTAGCGCTT-3′, SNX2 g#1 5′-CAGCGGAGGAGGTTCCCTCT-3′, SNX2 g#2 5′-GTCCTCCAGATCCTCAAAGT-3′, SNX5 g#1 5′-ACGAGGAGGCCGCCAACCGC-3′, SNX5 g#2 5′-GCGACACGGACGGGAAGCAA-3′, SNX6 g#1 5′-GCGGAGAACACCCACCATCA-3′, SNX6 g#2 5′- ATGATGGTGGGTGTTCTCCG-3′.

For dual CRISPRi constructs, dual sgRNA with SNX1 g#1 and SNX2 g#1 or SNX5 g#1 and SNX6 g#1 or controls (Ctrl neg g#1 and Ctrl neg g#2) were spaced by two BsmBI sites, annealed and cloned into BsmBI-digested pLentiCRISPRi-v3 vector. Then, a sgRNA scaffold CR3/mouse U6 promoter insert was digested with BsmBI and subsequently ligated into BsmBI-digested pLentiCRISPRi-v3 containing the dual sgRNA.

Human SNX1, SNX5 and SNX6 cDNA were amplified by RT-PCR using the SuperScript III (Invitrogen) from mRNAs of A549 cells using the following primers: SNX1-forward 5′-TAAGCGGCCGCATGGCGTCGGGTGGTGGTGG -3′; SNX1-reverse 5′-AATTAATTAAGTCGACTTAGGAGATGGCCTTTGCCTCAGG-3′; SNX5-forward 5′-GGGCGGCCGCATGGCCGCGGTTCCCGAGTTGCTGC-3′; SNX5-reverse 5′-AATTAATTAAGTCGACTCAGTTATTCTTGAACAAGCTAAT-3′; SNX6-forward 5′-GTGGATCCATGATGGAAGGCCTGGACGACGGCCCGGAC-3′; SNX6-reverse 5′-ACCTCGAGTCATGTGTCTCCATTTAACACTGCC-3′ and were cloned using NotI/SalI for SNX1 and SNX5, BamHI/XhoI for SNX6 in pRRL.sin.cPPT.SFFV/IRES-puro.WPRE.

The PX domain of SNX1, SNX5, and SNX6 were PCR-amplified from the aforementioned pRRL.sin.cPPT.SFFV/IRES-puro.WPRE LV vector plasmids and cloned into pMAL-p4X expression vectors (New England Biolabs) (previously modified using BamHI and HindIII sites to add one KpnI restriction site), using KpnI and HindIII cloning sites. The pMAL-PX-SNX5-F136A was obtained by site-directed mutagenesis (overlap-extension PCR).

The PX-SNX5-beta strands NCOA7-AS CDS was PCR-amplified using an overlapping strategy to generate the chimeric protein and cloned into pET-30 Ek/LIC expression vectors (Novagen) using KpnI and SalI cloning sites. The insert is in frame with the S- and His-tags and a sequence encoding the TEV protease cleavage site was inserted upstream of the chimera coding region to enable tag removal during the protein purification process. All plasmid constructs were verified by Sanger sequencing.

### Cell lines, LV vector production, and transduction

Human cell line U87-MG (ARP-2188) were obtained from the AIDS reagent program. Madin-Darby canine kidney (MDCK), A549 cells, and HEK293T were gifts from Prof. W. Barclay (Imperial College London) and Prof. M. Malim (King’s College London), respectively. These cell lines were maintained in Dulbecco's Modified Eagle Medium (DMEM) (Gibco) supplemented with 10% fetal bovine serum and 100 μg/ml penicillin and 100 units/ml streptomycin (Thermo Fisher Scientific). LV stocks were produced as described previously using polyethylenimine (14), either in HEK293T or in HEK293T LentiX (Clontech). Transduction with LVs was performed by incubating cells for 8 h prior to changing media for fresh media. Puromycin (1 μg/ml (Sigma-Aldrich)) and Blasticidin (10 μg/ml (InvivoGen) selections were performed 24 h posttransduction.

### IAV production and infection

Nanoluciferase-coding A/WSN/33 (H1N1) and A/Victoria/3/75 (H3N2) (14, 53) were produced as previously described (14). Briefly, the eight Pol I/PolII plasmids (0.5 μg each) for WSN rescue (a kind gift from Nadia Naffakh and Sandie Munier, Institut Pasteur Paris) or the eight Pol I plasmids (0.5 μg each) and four rescue plasmids (0.32 μg of PB1, PB2, PA and 0.64 μg NP plasmids) for Victoria virus rescue were cotransfected into HEK293T cells in 6-well plate format using Lipofectamine 3000 (Thermo Fisher Scientific). After 24 h, the cells were detached and cocultured with MDCK cells in 25 ml flasks for 8 h in DMEM containing 10% serum. The medium was then replaced with serum-free medium containing 1 μg/ml of L-1-p-Tosylamino-2-phenylethyl chloromethylketone-treated trypsin. Supernatants were used for virus amplification on MDCK cells. Viral stocks were titrated by plaque assays on MDCK cells.

For IAV single cycle infection, A549 cells stably expressing the constructs of interest were infected at an MOI of 0.5 for A/Victoria/3/75 (H3N2) and at an MOI of 0.25 for A/WSN/1933 for 1 h in serum-free DMEM. The media was then replaced with DMEM containing 10% serum, and 8 h postinfection, cells were washed with PBS and lysed in passive lysis buffer (Promega). Levels of infection were measured using the Nano-Glo Luciferase Assay System (Promega).

### Growth curve experiments

A549 stably expressing the constructs of interest were infected in triplicate in 12-well plates for 1 h at an MOI of 0.005 with A/WSN/1933. Cells were then washed with PBS and incubated in 1 ml of serum-free DMEM containing 1 μg/ml of L-1-p-Tosylamino-2-phenylethyl chloromethylketone-treated trypsin. Samples were collected at 24-, 48-, 72-, and 96 h postinfection and were titrated on MDCK cells by plaque assays.

### Coimmunoprecipitations

U87-MG cells or A549 cells were transduced to stably express single or triple (3 x) FLAG-tagged -NCOA7-AS, or mutants, -Firefly or -E2-Crimson. A total of 25 million cells were lysed in lysis buffer (20 mM Hepes-NaOH pH 7.5; 150 mM NaCl; 1% NP-40, and protease inhibitor cocktail (Roche)). Lysates were clarified by centrifugation at 1000*g* for 10 min at 4 °C. One fraction of cell lysates, corresponding to the whole-cell lysate was collected at this stage to serve as controls for protein input (10%) and the rest was incubated with FLAG-magnetic beads (Thermo Fisher Scientific) for 4 h at 4 °C. The beads were washed 5 times in lysis buffer and the immunoprecipitated proteins eluted using 150 μg/ml FLAG peptide (Sigma-Aldrich) in lysis buffer for 2 h at 4 °C.

### Mass spectrometry analysis

Sample digestion and peptides analysis were essentially performed as previously described (54). Briefly, purified proteins were loaded on an SDS-PAGE gel. After short migration, each sample was excised in one band. Proteins were digested using trypsin, and obtained peptides were analyzed online by nano-flow HPLC-nanoelectrospray ionization using a Qexactive HF mass spectrometer (Thermo Fisher Scientific) coupled to a nano-LC system (U3000-RSLC, Thermo Fisher Scientific). Desalting and preconcentration of samples were performed on-line on a Pepmap precolumn (0.3 × 10 mm; Dionex). A gradient consisting of 0 to 40% B in A for 120 min (A: 0.1% formic acid, 2% acetonitrile in water, and B: 0.1% formic acid in 80% acetonitrile) at 300 nl/min, was used to elute peptides from the capillary reverse-phase column (0.075 × 500 mm, Pepmap, Dionex). Data were acquired using Xcalibur software (version 4.1). A cycle of one full-scan mass spectrum (375–1500 m/z) at a resolution of 60,000 (at 200 m/z), followed by 12 data-dependent MS/MS spectra (at a resolution of 30,000, isolation window 1.2 m/z) was repeated continuously throughout the nanoLC separation.

Raw data analysis was performed using the MaxQuant software (version 1.5.5.1) with standard settings. Used database consist of human entries from Uniprot (reference proteome UniProt 2019_03) and 250 contaminants (MaxQuant contaminant database). Statistical analysis was performed using Perseus (v1.6.1.1). Intensity values were transformed in log2 scale and protein were filtered for at least three valid values in one of the experimental groups (NCOA7-AS or Control). Missing values were imputed by normal distribution (default parameter, assuming that most missing values are missing not at random and represent low abundance measurements). To highlight the proteins most abundant in NCOA7-AS *versus* Control, we used the built-in Two-sample test function in Perseus using standard parameters (Student’s *t* test, permutation base FDR, FDR of 5% and S0 of 0.1).

### Immunoblot analysis

Cells were washed with PBS and were lysed in sample buffer (10 mM Tris–HCl pH 7.6, 150 mM NaCl, 1% Triton X100, 1 mM EDTA, 0.1% deoxycholate, 2% SDS, 5% Glycerol, 100 mM DTT, and 0.02% bromophenol blue). Cell lysates were resolved by SDS-PAGE and analyzed by immunoblotting using primary antibodies specific for FLAG (M2, Sigma-Aldrich; 1/1000), SNX1 (10304-1-AP, Proteintech; 1/1000), SNX2 (611,308, BD Biosciences; 1/1000), SNX5 (17918-1-AP, Proteintech; 1/1000), SNX6 (10114-1-AP, Proteintech; 1/300), ATP6V1A (17115-1-AP, Proteintech; 1/1000), ATP6V1B2 (15097-1-AP, Proteintech; 1/1000), ATP6V1E1 (15280-1-AP, Proteintech; 1/1000), actin (A1978, Sigma-Aldrich; 1/5000), followed by secondary horseradish peroxidase (HRP)-conjugated anti-mouse or anti-rabbit immunoglobulin antibodies or primary GAPDH antibody coupled to HRP GAPDH-HRP antibody (G9295, Sigma-Aldrich, one-fifth000) and primary FLAG antibody coupled to HRP FLAG-HRP antibody (G8592, Sigma-Aldrich, 1/10,000). Chemiluminescence was then measured using the Clarity ECL Western Blotting Substrate, Bio-Rad and a ChemiDoc gel imaging system (Bio-Rad).

### Lysosomal compartment expansion and acidification

Transduced A549 cells were incubated with 0.1 μM LysoSensor Green DND-189 (Thermo Fisher Scientific) or with 0.1 μM LysoTracker Green DND-26 (Thermo Fisher Scientific) for 1 h at 37 °C. Cells were then washed, trypsinized and analyzed immediately by flow cytometry on a BD LSRFortessa.

### Protein expression and purification

#### GST-tagged proteins

The recombinant plasmids pET-30 Ek/LIC expressing GST, NCOA7-AS-WT-GST and mutants, NCOA7-AS-TLDc-GST, NCOA7-AS-NTD-GST were transformed in an *E. coli* BL21 (DE3) strain resistant to Phage T1 (New England Biolabs) carrying pRARE2. One colony was used to inoculate an overnight culture of 125 ml Lysogeny broth (LB) medium supplemented with kanamycin (50 μg/ml) and chloramphenicol (30 μg/ml). This culture was diluted in 2.5 L of LB medium supplemented with the two antibiotics. The cells were grown at 37 °C to an optical density at 600 nm of 0.8, then a 30 min cold shock on ice was performed and protein expression was induced with 1 mM Isopropyl-β-D-thiogalactoside (IPTG) and the culture was grown overnight at 16 °C (except for GST for which bacteria were grown 3 h at 37 °C after adding IPTG). The cells were harvested by centrifugation at 8,200*g* for 20 min and resuspended in 30 ml of buffer A (50 mM Tris–HCl pH 8, 400 mM NaCl, 5 mM β-mercaptoethanol, 40 mM imidazole, 10% glycerol and 1 mM benzamidine). The cells were disrupted by sonication and cell debris were removed by centrifugation at 28,000 *g* for 60 min. The supernatant was loaded at 4 °C on Ni–NTA sepharose beads (GE17-5318-01, Sigma-Aldrich) previously equilibrated with buffer A. The beads were washed once with buffer A and twice with buffer B (50 mM Tris–HCl pH 8, 1 M NaCl, 5 mM β-mercaptoethanol, 40 mM imidazole and 10% glycerol) and elution was performed with buffer E (50 mM Tris–HCl pH 8, 600 mM NaCl, 5 mM β-mercaptoethanol, 250 mM imidazole and 10% glycerol). The eluted protein was dialyzed (dialysis-bag cutoff 12–15 kDa) against 1 L dialysis buffer D (50 mM Tris–HCl pH 8, 600 mM NaCl, 5 mM β-mercaptoethanol, 10% glycerol) overnight at 4 °C. After dialysis, the proteins were concentrated to 5 mg/ml using a Vivaspin column (10 kDa cutoff, Sartorius), loaded onto a size-exclusion chromatography column (Superdex 200 increase 10/300 Gl, GE Healthcare) and eluted with buffer F (50 mM Tris–HCl pH 8, 600 mM NaCl, 5 mM β-mercaptoethanol and 10% glycerol). Aliquots of purified GST-tagged proteins were snap frozen in liquid nitrogen and stored at −80 °C.

### MBP-tagged proteins

The recombinant plasmids pMAL-p4X expressing MBP-PX-SNX1, 5, and six were transformed in an *E. coli* BL21 (DE3) strain resistant to Phage T1 (New England Biolabs) carrying pRARE2. One colony was used to inoculate an overnight culture of 125 ml LB medium supplemented with kanamycin (50 μg/ml) and chloramphenicol (30 μg/ml). This culture was diluted in 2.5 L of LB medium supplemented with the two antibiotics. The cells were grown at 37 °C to an optical density at 600 nm of 0.8, then a 60 min cold shock on ice was performed, protein expression was induced with 1 mM IPTG and the culture was grown overnight at 16 °C. The cells were harvested by centrifugation at 8200*g* for 20 min and resuspended in 30 ml of buffer A (50 mM Tris–HCl pH 8, 400 mM NaCl, 5 mM β-mercaptoethanol, 10% glycerol and 1 mM benzamidine). The cells were disrupted by sonication and cell debris were removed by centrifugation at 28,000 *g* for 60 min. The supernatant was loaded at 4 °C on Amylose resin previously equilibrated with buffer A. The beads were washed once with buffer A and twice with buffer B (50 mM Tris–HCl pH 8, 1 M NaCl, 5 mM β-mercaptoethanol, and 10% glycerol) and elution was performed with buffer E (50 mM Tris–HCl pH 8, 600 mM NaCl, 5 mM β-mercaptoethanol, 20 mM maltose and 10% glycerol). The eluted protein was dialyzed (dialysis-bag cutoff 12–15 kDa) against 1 L dialysis buffer D (50 mM Tris–HCl pH 8, 600 mM NaCl, 5 mM β-mercaptoethanol and 10% glycerol) overnight at 4 °C. After dialysis, the proteins were concentrated to 5 mg/ml using a Vivaspin column (10 kDa cutoff, Sartorius), loaded onto a size-exclusion chromatography column (Superdex 200 increase 10/300 Gl, GE Healthcare) and eluted with buffer F (50 mM Tris–HCl pH 8, 600 mM NaCl, 5 mM β-mercaptoethanol and 10% glycerol). Aliquots of purified MBP-tagged proteins were snap frozen in liquid nitrogen and stored at −80 °C.

## GST pull-down

Recombinant GST-tagged proteins (14–35 μg) were incubated 2 h at 4 °C with glutathione agarose beads prewashed in pull-down assay buffer (50 mM Tris–HCl pH 8, 600 mM NaCl, 5 mM β-mercaptoethanol and 10% glycerol). Beads were then washed 3 times with pull-down assay buffer, then incubated for 2 h at 4 °C with 85 μg of recombinant MBP-tagged protein preincubated with glutathione agarose beads for 2 h at 4 °C. Beads were finally washed 3 times with pull-down assay buffer before adding 25 μl of Laemmli 2X. Samples were resolved by SDS-PAGE on a 10% acrylamide gel after 10 min boiling. Immunoblotting was performed by incubation with a primary GST antibody coupled to HRP (A7340, Sigma-Aldrich) and an MBP antibody (E8032, NEB) followed by an HRP-conjugated secondary antibody. Bioluminescence was then measured (Clarity ECL Western Blotting Substrate, Bio-Rad) using a ChemiDoc system (Bio-Rad).

### *K*d determination

Recombinant MBP-PX-SNX5 and MBP-PX-SNX5-F136A were dialyzed overnight at 4 °C against 1 L of PBS-10% glycerol. Concentrated proteins (0.38 μM to 250 μM) were mixed with 57 nM final FITC conjugated NCOA7-AS peptide (NH2-RLPLDIQIFY CARPDEEPFV KIITVEEAKR R-COOH) before loading on a Fluidity One chip. Hydrodynamic radius were given by Fluidity One-W apparatus (Fluidic Analytics). The data were analyzed with GraphPad prism version 11.02 and using the “One site--specific binding” function. The apparent *K*d were calculated with the following equation: Y = Bmax∗X/(*K*d + X), where Bmax is the number of accessible binding site and X the ligand concentration. The given *K*d value is the mean of the *K*d values of each individual dataset and the variability is represented by the standard deviation.

### Expression and purification of the chimera PX-SNX5-beta strands NCOA7-AS

The recombinant plasmid pET-30-PX-SNX5-beta strands NCOA7-AS was transformed in an *E. coli* BL21 (DE3) strain resistant to Phage T1 (New England Biolabs) carrying pRARE2. One colony was used to inoculate an overnight culture of 125 ml LB medium supplemented with kanamycin (50 μg/ml) and chloramphenicol (30 μg/ml). This culture was diluted in 2.5 L of LB medium supplemented with the two antibiotics. The cells were grown at 37 °C to an optical density at 600 nm of 0.8, then protein expression was induced with 1 mM IPTG and the culture was grown 3 h at 37 °C. The cells were harvested by centrifugation at 8200*g* for 20 min and resuspended in 30 ml of buffer A (50 mM Tris–HCl pH 8, 400 mM NaCl, 5 mM β-mercaptoethanol, 40 mM imidazole, 10% glycerol and 1 mM benzamidine). The cells were disrupted by sonication and cell debris were removed by centrifugation at 28,000*g* for 60 min. The supernatant was loaded at 4 °C on Ni–NTA sepharose beads previously equilibrated with buffer A. The beads were washed once with buffer A and twice with buffer B (50 mM Tris–HCl pH 8, 1 M NaCl, 5 mM β-mercaptoethanol, 40 mM imidazole and 10% glycerol) and elution was performed with buffer E (50 mM Tris–HCl pH 8, 200 mM NaCl, 5 mM β-mercaptoethanol, 250 mM imidazole and 10% glycerol). The eluted protein was incubated with His-tagged TEV protease purified in our laboratory in a 1:100 (w:w) ratio; the cleavage reaction was performed during dialysis (dialysis-bag cutoff 12–15 kDa) against 1 L dialysis buffer D (50 mM Tris–HCl pH 8, 200 mM NaCl, and 5 mM β-mercaptoethanol) overnight at 4 °C. After dialysis, the proteins were centrifuged for 20 min at 28,000*g* and the supernatant was loaded again at 4 °C on Ni–NTA sepharose beads equilibrated with buffer D. The proteins without the His-tag were collected in the flow-through, concentrated to 5 mg/ml using a Vivaspin column (10 kDa cutoff, Sartorius), loaded onto a size-exclusion chromatography column (Superdex 75 Increase 10/300 Gl, GE Healthcare), and eluted with buffer F (50 mM Tris–HCl pH 8, 100 mM NaCl and 5 mM β-mercaptoethanol). Following this protocol, 3.5 mg pure protein was obtained from 1 L culture.

### Protein crystallization

Crystals of the trigonal form were obtained in sitting drops using the SwissCi (MRC) 96 well plate mixing 0.6 μl of protein solution concentrated to 17 mg/ml with 0.6 μl of reservoir solution made of 0.2 M Calcium acetate hydrate, 0.1 M Sodium Cacodylate pH6.5, and 18% w/v polyethylene glycol 8000 and equilibrated against a reservoir volume of 50 μl at 18 °C. The monoclinic crystals were obtained similarly by mixing 0.6 μl of protein solution concentrated to 17 mg/ml with 0.6 μl of reservoir solution made of 0.1 M sodium citrate tribasic dihydrate pH 5.0, 30% v/v jeffamine ED-2001 pH 7.0 and were not cryoprotected before being cryocooled.

### X-ray data collection and processing

Data collection was performed on the PXIII-X06DA beamline at the Swiss Light Source, Paul Scherrer Institute. Data were processed and scaled with the XDS package (55). The phase problem was solved by molecular replacement using Phaser (56) and the Phenix package (57), and the structure of the human SNX5 PX domain (5wy2) (41) as a search template. The structures were further manually rebuilt with Coot (58) and refined with the Phenix package (57).

### Statistical analyses

Statistical analyses were performed using GraphPad Prism software using the indicated tests. *p*-values are indicated as the following: ns = not significant; *p* < 0.05 = ∗; *p* < 0.01 = ∗∗; *p* < 0.001 = ∗∗∗; and *p* < 0.0001 = ∗∗∗∗.

## Data availability

All data are present within this article and/or available upon request to the corresponding authors. Structural data were deposited at the Protein Data Bank under the two following accession numbers: 9IAE and 9IGY. The mass spectrometry proteomics data have been deposited to the ProteomeXchange Consortium *via* the PRIDE (59) partner repository with the dataset identifier PXD080054.

## Supporting information

This article contains supporting information.

## Conflict of interest

The authors declare that they have no conflicts of interest with the contents of this article.

## References

[1] Dou D., Revol R., Östbye H., Wang H., Daniels R. (2018). Influenza A virus cell entry, replication, virion assembly and movement. Front. Immunol..

[2] Matlin K.S., Reggio H., Helenius A., Simons K. (1981). Infectious entry pathway of influenza virus in a canine kidney cell line. J. Cell Biol..

[3] McKellar J., Cadènes J., Gracia, F.G. de, Aubé C., Valadão A.L.C., Tauziet M. (2025). Human MX1 orchestrates the cytoplasmic sequestration of neosynthesized influenza A virus vRNPs. Proc. Natl. Acad. Sci. U. S. A..

[4] Cotter K., Stransky L., McGuire C., Forgac M. (2015). Recent insights into the structure, regulation, and function of the V-ATPases. Trends Biochem. Sci..

[5] Pinto L.H., Holsinger L.J., Lamb R.A. (1992). Influenza virus M2 protein has ion channel activity. Cell.

[6] Pinto L.H., Lamb R.A. (2006). The M2 proton channels of influenza A and B viruses. J. Biol. Chem..

[7] Stauffer S., Feng Y., Nebioglu F., Heilig R., Picotti P., Helenius A. (2014). Stepwise priming by acidic pH and a high K+ concentration is required for efficient uncoating of influenza A virus cores after penetration. J. Virol..

[8] Wharton S.A., Belshe R.B., Skehel J.J., Hay A.J. (1994). Role of virion M2 protein in influenza virus uncoating: specific reduction in the rate of membrane fusion between virus and liposomes by amantadine. J. Gen. Virol..

[9] Helenius A. (1992). Unpacking the incoming influenza virus. Cell.

[10] Zhirnov O.P. (1990). Solubilization of matrix protein M1/M from virions occurs at different pH for orthomyxo- and paramyxoviruses. Virology.

[11] McKellar J., Rebendenne A., Wencker M., Moncorgé O., Goujon C. (2021). Mammalian and Avian host cell influenza A restriction factors. Viruses.

[12] Villalón-Letelier F., Brooks A.G., Saunders P.M., Londrigan S.L., Reading P.C. (2017). Host cell restriction factors that limit influenza A infection. Viruses.

[13] Yu L., Croze E., Yamaguchi K.D., Tran T., Reder A.T., Litvak V. (2015). Induction of a unique isoform of the *NCOA7* oxidation resistance gene by interferon β-1b. J. Interferon Cytokine Res..

[14] Doyle T., Moncorgé O., Bonaventure B., Pollpeter D., Lussignol M., Tauziet M. (2018). The interferon-inducible isoform of NCOA7 inhibits endosome-mediated viral entry. Nat. Microbiol..

[15] Khan H., Winstone H., Jimenez-Guardeño J.M., Graham C., Doores K.J., Goujon C. (2021). TMPRSS2 promotes SARS-CoV-2 evasion from NCOA7-mediated restriction. PLoS Pathog..

[16] Wickenhagen A., Sugrue E., Lytras S., Kuchi S., Noerenberg M., Turnbull M.L. (2021). A prenylated dsRNA sensor protects against severe COVID-19. Science.

[17] Castroflorio E., Hoed, J. den, Svistunova D., Finelli M.J., Cebrian-Serrano A., Corrochano S. (2021). The Ncoa7 locus regulates V-ATPase formation and function, neurodevelopment and behaviour. Cell. Mol. Life Sci..

[18] Merkulova M., Păunescu T.G., Azroyan A., Marshansky V., Breton S., Brown D. (2015). Mapping the H+ (V)-ATPase interactome: identification of proteins involved in trafficking, folding, assembly and phosphorylation. Sci. Rep..

[19] Blaise M., Alsarraf H.M., Wong J.E., Midtgaard S.R., Laroche F., Schack L. (2012). Crystal structure of the TLDc domain of oxidation resistance protein 2 from zebrafish. Proteins.

[20] Finelli M.J., Oliver P.L. (2017). TLDc proteins: new players in the oxidative stress response and neurological disease. Mamm. Genome.

[21] Eaton A.F., Brown D., Merkulova M. (2021). The evolutionary conserved TLDc domain defines a new class of (H+)V-ATPase interacting proteins. Sci. Rep..

[22] Burd C., Cullen P.J. (2014). Retromer: a master conductor of endosome sorting. Cold Spring Harbor Perspect. Biol..

[23] Simonetti B., Paul B., Chaudhari K., Weeratunga S., Steinberg F., Gorla M. (2019). Molecular identification of a BAR domain-containing coat complex for endosomal recycling of transmembrane proteins. Nat. Cell Biol..

[24] Wang R., Qin Y., Xie X.-S., Li X. (2022). Molecular basis of mEAK7-mediated human V-ATPase regulation. Nat. Commun..

[25] Burda P., Padilla S.M., Sarkar S., Emr S.D. (2002). Retromer function in endosome-to-golgi retrograde transport is regulated by the yeast Vps34 PtdIns 3-kinase. J. Cell Sci..

[26] Cheever M.L., Sato T.K., Beer, T. de, Kutateladze T.G., Emr S.D., Overduin M. (2001). Phox domain interaction with PtdIns(3)P targets the Vam7 t-SNARE to vacuole membranes. Nat. Cell Biol..

[27] Gillooly D.J., Raiborg C., Stenmark H. (2003). Phosphatidylinositol 3-phosphate is found in microdomains of early endosomes. Histochem. Cell Biol..

[28] Yu J.W., Lemmon M.A. (2001). All Phox Homology (PX) domains from Saccharomyces cerevisiae specifically recognize phosphatidylinositol 3-Phosphate. J. Biol. Chem..

[29] Chandra M., Chin Y.K.-Y., Mas C., Feathers J.R., Paul B., Datta S. (2019). Classification of the human phox homology (PX) domains based on their phosphoinositide binding specificities. Nat. Commun..

[30] Carlton J., Bujny M., Peter B.J., Oorschot V.M.J., Rutherford A., Mellor H. (2004). Sorting nexin-1 mediates tubular endosome-to-TGN transport through coincidence sensing of high- curvature membranes and 3-phosphoinositides. Curr. Biol..

[31] Frost A., Unger V.M., Camilli P.D. (2009). The BAR domain superfamily: membrane-molding macromolecules. Cell.

[32] Weering, J.R.T. van, Verkade P., Cullen P.J. (2010). SNX-BAR proteins in phosphoinositide-mediated, tubular-based endosomal sorting. Semin. Cell Dev. Biol..

[33] Wassmer T., Attar N., Bujny M.V., Oakley J., Traer C.J., Cullen P.J. (2006). A loss-of-function screen reveals SNX5 and SNX6 as potential components of the mammalian retromer. J. Cell Sci..

[34] Kvainickas A., Jimenez-Orgaz A., Nägele H., Hu Z., Dengjel J., Steinberg F. (2017). Cargo-selective SNX-BAR proteins mediate retromer trimer independent retrograde transport. J. Cell Biol..

[35] Simonetti B., Danson C.M., Heesom K.J., Cullen P.J. (2017). Sequence-dependent cargo recognition by SNX-BARs mediates retromer-independent transport of CI-MPR. J. Cell Biol..

[36] Yong X., Zhao L., Deng W., Sun H., Zhou X., Mao L. (2020). Mechanism of cargo recognition by retromer-linked SNX-BAR proteins. PLoS Biol..

[37] Dong X., Yang Y., Zou Z., Zhao Y., Ci B., Zhong L. (2021). Sorting nexin 5 mediates virus-induced autophagy and immunity. Nature.

[38] Elwell C.A., Czudnochowski N., Dollen J.V., Johnson J.R., Nakagawa R., Mirrashidi K. (2017). Chlamydia interfere with an interaction between the mannose-6-phosphate receptor and sorting nexins to counteract host restriction. eLife.

[39] Mirrashidi K.M., Elwell C.A., Verschueren E., Johnson J.R., Frando A., Von Dollen J. (2015). Global mapping of the Inc-human interactome reveals that retromer restricts chlamydia infection. Cell Host Microbe.

[40] Paul B., Kim H.S., Kerr M.C., Huston W.M., Teasdale R.D., Collins B.M. (2017). Structural basis for the hijacking of endosomal sorting nexin proteins by Chlamydia trachomatis. eLife.

[41] Sun Q., Yong X., Sun X., Yang F., Dai Z., Gong Y. (2017). Structural and functional insights into sorting nexin 5/6 interaction with bacterial effector IncE. Signal. Transduct. Target. Ther..

[42] Arnaud-Arnould M., Tauziet M., Moncorgé O., Goujon C., Blaise M. (2021). Crystal structure of the TLDc domain of human NCOA7-AS. Acta Crystallogr. F Struct. Biol. Commun..

[43] Arosio P., Mu¨ller T., Rajah L., Yates E.V., Aprile F.A. (2016). Microfluidic diffusion analysis of the sizes and interactions of proteins under native solution conditions. ACS Nano.

[44] Herling T.W., O’Connell D.J., Bauer M.C., Persson J., Weininger U., Knowles T.P.J. (2016). A microfluidic platform for real-time detection and quantification of protein-ligand interactions. Biophys. J..

[45] O’Mahoney C., Watt I., Fiedler S., Devenish S., Srikanth S., Justice E. (2024). Microfluidic diffusional Sizing (MDS) measurements of secretory neutralizing antibody affinity against SARS-CoV-2. Ann. Biomed. Eng..

[46] Wright M.A., Aprile F.A., Bellaiche M.M.J., Michaels T.C.T., Mu¨ller T. (2018). Cooperative assembly of Hsp70 subdomain clusters. Biochemistry.

[47] Maxson M.E., Grinstein S. (2014). The vacuolar-type H+-ATPase at a glance - more than a proton pump. J. Cell Sci..

[48] Khan, Md.M., Lee S., Couoh-Cardel S., Oot R.A., Kim H., Wilkens S. (2022). Oxidative stress protein Oxr1 promotes V-ATPase holoenzyme disassembly in catalytic activity-independent manner. EMBO J..

[49] Oot R.A., Wilkens S. (2024). Human V-ATPase function is positively and negatively regulated by TLDc proteins. Structure.

[50] Bonaventure B., Rebendenne A., Valadão A.L.C., Arnaud-Arnould M., Gracias S., Gracia, F.G. de (2022). The DEAD box RNA helicase DDX42 is an intrinsic inhibitor of positive-strand RNA viruses. EMBO Rep..

[51] McKellar J., Arnaud-Arnould M., Chaloin L., Tauziet M., Arpin-André C., Pourcelot O. (2023). An evolutionarily conserved N-terminal leucine is essential for MX1 GTPase antiviral activity against different families of RNA viruses. J. Biol. Chem..

[52] Alerasool N., Segal D., Lee H., Taipale M. (2020). An efficient KRAB domain for CRISPRi applications in human cells. Nat. Methods.

[53] Diot C., Fournier G., Santos M.D., Magnus J., Komarova A., Werf S.V.D. (2016). Influenza A virus polymerase recruits the RNA helicase DDX19 to promote the nuclear export of viral mRNAs. Sci. Rep..

[54] Sirvent A., Vigy O., Orsetti B., Urbach S., Roche S. (2012). Analysis of SRC oncogenic signaling in colorectal cancer by stable isotope labeling with heavy amino acids in mouse xenografts. Mol. Cell Proteomics.

[55] Kabsch W. (2010). XDS. Acta Crystallogr. D Biol. Crystallogr..

[56] McCoy A.J. (2007). Solving structures of protein complexes by molecular replacement with *phaser*. Acta Crystallogr. D Biol. Crystallogr..

[57] Liebschner D., Afonine P.V., Baker M.L., Bunkóczi G., Chen V.B., Croll T.I. (2019). Macromolecular structure determination using X-rays, neutrons and electrons: recent developments in *Phenix*. Acta Crystallogr. D Struct. Biol..

[58] Casañal A., Lohkamp B., Emsley P. (2020). Current developments in *coot* for macromolecular model building of electron Cryo-microscopy and crystallographic data. Protein Sci..

[59] Perez-Riverol Y., Bandla C., Kundu D.J., Kamatchinathan S., Bai J., Hewapathirana S. (2024). The PRIDE database at 20 years: 2025 update. Nucleic Acids Res..

