# Supplementary material for: SNX-BAR proteins 5 and 6 are required for NCOA7-AS antiviral activity against influenza A virus
Source: J Biol Chem. 2026 Jul 28;302(9):113372. doi: 10.1016/j.jbc.2026.113372 (PMC13543948; doi:10.1016/j.jbc.2026.113372)
Supplement: Supplementary file 1 [file mmc1.docx]

**Supplementary Figures**

**Supplementary Figure 1. A.** AlphaFold prediction of NCOA7-AS with the N-terminal domain (NTD-amino-acids 1-53) colored in green and the C-terminal TLDc colored in purple. The NTD contains two antiparallel β-strands and a small α-helix. **B.** PSIPRED predictions, highlighting the presence of a ΦxΩxΦ (black box) in the first β-strand of NCOA7-AS.

**Supplementary Figure 2. A.** The recombinant PX-SNX5-NCOA7-AS chimeric protein was produced in *Escherichia coli* and purified. The elution profile on Superdex 75 column is shown with the different fractions collected that have been analyzed by Coomassie staining. **B.** Comparison of the interface of PX-SNX5/NCOA7-AS with all the previously solved structure of PX-SNX5 bound to other binding partners. PX-SNX5::Sema4C (PDB: 6n5z), PX-SNX5::CI-MPR (PDB: 6n5y) and PX-SNX5::IncE (PDB: 5wy2).
