# Supplementary material for: SNX-BAR proteins 5 and 6 are required for NCOA7-AS antiviral activity against influenza A virus
Source: J Biol Chem. 2026 Jul 28;302(9):113372. doi: 10.1016/j.jbc.2026.113372 (PMC13543948; doi:10.1016/j.jbc.2026.113372)
Supplement: Supplementary file 2 [file mmc3.docx]

**Supplementary Table 2.** X-ray data collection and refinement statistics

|  | **Nt-NCOA7-PX-SNX5**  **Form I** | **Nt-NCOA7-PX-SNX5**  **Form II** |
| --- | --- | --- |
| **PDB deposition code** | 9IAE | 9IGY |
| **Wavelength (Å)** | 1 | 1 |
| **Resolution range** | 48.1 - 2.3 (2.38 - 2.3) | 46.1 - 2.3 (2.36 - 2.3) |
| **Space group** | *P* 3_2_ 2 1 | *P* 1 2_1_ 1 |
| **Unit cell** | 63.13 63.13 101.51  90 90 120 | 46.9 76.99 110.85  90 100.181 90 |
| **Total reflections** | 210470 (25566) | 230324 (29031) |
| **Multiplicity** | 19.6 (20.14) | 6.8 (7.0) |
| **Completeness (%)** | 98.6 (100.00) | 97.6 (99.9) |
| **Mean I/sigma(I)** | 16.02 (2.23) | 10.98 (1.61) |
| **Wilson B-factor** | 37.3 | 38.6 |
| **R-meas (%)** | 19.3 (166.3) | 16.2 (130.1) |
| **CC1/2** | 0.999 (0.755) | 0.997 (0.695) |
| **Reflections used in refinement** | 10727 (1332) | 33853 (2487) |
| **Reflections used for R-free** | 1076 (106) | 2015 (253) |
| **R-work** | 0.201 (0.247) | 0.227 (0.289) |
| **R-free** | 0.239 (0.346) | 0.262 (0.323) |
| **Number of non-hydrogen atoms** | 1595 | 5665 |
| **macromolecules** | 1434 | 5437 |
| **solvent** | 161 | 228 |
| **Protein residues** | 173 | 665 |
| **RMSD (bonds, Å)** | 0.006 | 0.002 |
| **RMSD (angles, °)** | 0.52 | 0.41 |
| **Ramachandran favored (%)** | 98.21 | 97.53 |
| **Ramachandran allowed (%)** | 1.79 | 2.47 |
| **Ramachandran outliers (%)** | 0.00 | 0.00 |
| **Rotamer outliers (%)** | 1.25 | 0.81 |
| **Clashscore** | 3.16 | 3.24 |
| **Average B-factor (Å^2^)** | 46.13 | 46.58 |
| **macromolecules** | 44.91 | 46.75 |
| **solvent** | 45.37 | 42.60 |

Statistics for the highest-resolution shell are shown in parentheses.
